# Possible long COVID healthcare pathways: a scoping review

**DOI:** 10.1186/s12913-022-08384-6

**Published:** 2022-08-23

**Authors:** Sarah Wolf, Ingrid Zechmeister-Koss, Judit Erdös

**Affiliations:** Austrian Institute for Health Technology Assessment GmbH, Vienna, Austria

**Keywords:** Long COVID, Healthcare pathways, Recommendations, Guidelines, Scoping review

## Abstract

**Background:**

Individuals of all ages and with all degrees of severity of the coronavirus disease (COVID) can suffer from persisting or reappearing symptoms called long COVID. Long COVID involves various symptoms, such as shortness of breath, fatigue, or organ damage. The growing number of long COVID cases places a burden on the patients and the broader economy and, hence, has gained more weight in political decisions. This scoping review aimed to give an overview of recommendations about possible long COVID healthcare pathways and requirements regarding decision-making and communication for healthcare professionals.

**Methods:**

A systematic search in four databases and biweekly update-hand searches were conducted. In addition to guidelines and reviews, expert opinions in consensus statements or clinical perspectives were also considered. Data were systematically extracted and subsequently narratively and graphically summarised.

**Results:**

Fourteen references, five guidelines, four reviews, one consensus paper, and four clinical perspectives were included. The evidence recommended that most long COVID-related healthcare should be in primary care. Patients with complex symptoms should be referred to specialized long COVID outpatient assessment clinics. In contrast, patients with one dominant symptom should be directed to the respective specialist for a second assessment. Depending on the patients’ needs, further referral options include, e.g. rehabilitation or non-medical health services. Self-management and good communication between healthcare professionals and patients are crucial aspects of the long COVID management recommendations.

**Conclusions:**

The quality of the included guidelines and reviews is limited in the methods applied due to the novelty of this topic and the associated urgency for research. Hence, an update review with more rigorous data is recommended. Furthermore, the systematic collection of real-world data on long COVID surveillance needs to be set up soon to gather further information on the duration and severity of long COVID and thereby facilitate long COVID care planning.

## Background

Long COVID patients experience various symptoms that can persist or reappear after a severe acute respiratory syndrome coronavirus 2 (SARS-CoV-2) infection, including fatigue, shortness of breath, high blood pressure, olfactory and gustatory disturbances, neurocognitive disorders or psychological complaints, such as anxiety and depression. In severe cases, organ damage, e.g., to the heart, lungs, or liver, may also occur [1]. Individuals of all ages and with all degrees of severity of the acute infection can suffer from long COVID that generally impacts the patients’ everyday functioning [2–4].

### Definitions

In December 2020, the National Institute for Health and Care Excellence (NICE) in the United Kingdom (UK) proposed a definition of long COVID, which includes all symptoms that occur after an acute SARS-CoV-2 infection and cannot be associated with any other cause. The definition distinguishes between different time points [5]:

- 4–12 weeks after the SARS-CoV-2 infection: “ongoing symptomatic COVID-19”
- > 12 weeks after the SARS-CoV-2 infection: “post-COVID-19 syndrome”

In October 2021, the World Health Organisation (WHO) published a clinical case definition of the post-COVID-19 condition, developed by Delphi methodology and following the NICE definition. Accordingly, post-COVID-19 includes individuals with a history of probable or confirmed SARS CoV-2 infection and symptoms that usually persist or reoccur 3 months from the onset of COVID-19 and cannot be explained by an alternative diagnosis [4].

### Diagnosis

Since January 2021, the ICD-10 catalogue of the WHO issued a separate diagnosis code for long COVID, namely U09.9 [6]. In addition to the single ICD-10 code for long COVID, it is recommended to list other specific (organ)diagnoses on an ICD-basis due to the tremendous variety in long COVID symptoms and the associated difficult diagnosis [7].

### Epidemiology and societal impact

Overall, a summary of 47 studies showed that five to 36% of the COVID-19 patient, of which the majority had not been hospitalized during the acute infection, and 32 to 78% of the mainly hospitalized COVID-19 patients suffered from persistent symptoms 1 to 3 months after the acute infection. After 3 to 6 months, the prevalence slightly decreased in the non-hospitalized patient group (2–21%) but increased in the hospitalized COVID-19 group (13–92%). After 6 months, 13 to 53% of the primarily non-hospitalized and up to 50 to 93% of the mainly hospitalized COVID-19 patients reported ongoing symptoms [8]. Due to the increasing number of new COVID-19 patients, the number of long COVID patients is expected to increase. Consequently, this trend places a burden on the patients and their families and the wider economy, particularly the workforce (e.g. increased sick leave durations or part-time workers) [2, 3, 8]. For this reason, long COVID care planning has gained more weight in political decisions.

This paper aims to provide a scoping review of possible long COVID healthcare pathways for adults and requirements regarding decision-making and communication for healthcare professionals. The presented results are deemed to support preparations for and adjustments in the long COVID healthcare planning.

## Methods

For this scoping review, the Preferred Reporting Items for Systematic Reviews and Meta-Analyses (PRISMA) statement was utilised as reporting guidance (without the quality appraisal section) [9].

### Literature search and selection

From 27th to 29th April 2021, a systematic literature search was performed in four databases (Cochrane, Embase, Medline, INAHTA). In addition, hand searches were conducted on a biweekly basis to gather further information (last hand search: 02.08.2021). More details on the systematic search strategy can be requested from the authors.

For the selection of the literature, inclusion and exclusion criteria were defined according to the PICO scheme. We included guidelines, reviews and expert opinions in the form of consensus statements and clinical perspectives from Europe, North America and Australia that presented recommendations about possible long COVID healthcare pathways for adult patients with symptoms lasting more than 4 weeks after the acute infection. All papers in English and German published before August 2021 were considered. The literature was reviewed independently by two authors (SW, JE). Differences were resolved through discussion and consensus or the involvement of a third person.

### Quality assessment and extraction of data

In this scoping review, no systematic quality assessment of the included guidelines or reviews was planned because most of the available references were conceptual and did not fit a classical quality evaluation. Data of included references were extracted into data extraction tables. Table 1 presents the characteristics of the included literature (authors, year, country funding, patient population, etc.). The results about the long COVID healthcare pathways were narratively summarized in different chapters: first point of services, possible referrals and further recommendations regarding decision-making and communication. In addition, the identified long COVID healthcare pathways were graphically summarized. The detailed extraction tables about the possible long COVID healthcare pathways can be requested from the authors.

**Table 1:** Characteristics of the included references

Authors, month/year, reference	Country (institute)	Evidence-base	Funding, CoI	Long COVID definition	Patient population
**(Living) guidelines**					
Shah et al. 12/2020 [5]	UK (NICE, RCGP, SIGN)	Consensus-based	NR	Symptoms for > 4 weeks after the acute infection	Pts. treated at the hospital & on community level (e.g. at home)
04/2021 [10]	UK (NHS)	Consensus-based	NR		
2021 [11]	USA (CDC)	NR	NR	NR	NR
Koczulla et al. 7/2021 [7]	GER	Consensus-based	NR	Symptoms for > 4 weeks after the acute infection	Pts. treated at the hospital & on community level (e.g. at home)
Rabady et al. 7/2021 [12]	AT	Consensus-based	**Funding**: NR **CoI**: None	Symptoms for > 4 weeks after the acute infection	Pts. treated at the hospital (except ICU) & on community level (e.g. at home)
**Reviews**					
03/2021 [2]	UK (NIHR)	Evidence-based	NR	NR	Pts. treated at the hospital & on community level (e.g. at home)
Parkin et al. 03/2021 [13]	UK	NR	**Funding**: Leed Clinical Commissioning Group, University of Lees Medical Research Council Confidence in Concept grant **CoI**: None	Symptoms for > 4 weeks after the acute infection	
Oronsky et al. 2021 [14]	USA	NR	NR	NR	NR
Rajan et al. 03/2021 [3]	European countries, e.g. UK, GER, IT, BE	NR	**Funding**: NIHR, ARC EM, Leicester Biomedical Research Centre **CoI**: Two authors reported CoI	Symptoms for > 6 weeks after the acute infection	NR
**Expert papers**					
**Consensus paper**					
Barker-Davis et al. 05/2020 [15]	UK	Consensus-based	**Funding**: None **CoI**: None	NR	Active pts. including military personnel & athletes
**Clinical perspectives**					
Greenhalgh et al. 2020 [16]	UK	Consensus-based	**Funding**: None **CoI**: None	Symptoms for > 4 weeks after the acute infection	Pts. treated at the hospital and on community level (e.g. at home)
Spruit et al. 2020 [17]	NL	Consensus-based	**Funding:** NR **CoI:** Three authors reported CoI	Symptoms for 4–12 weeks after the acute infection	Pts. treated at the hospital
Halle et al. 2021 [18]	GER	Consensus-based	**Funding:** NR **CoI:** None	NR	NR
Leo et al. 2020 [19]	GER	Consensus-based	**Funding:** NR **CoI:** None	NR	Pts. treated at the hospital and on community level (e.g. outpatient, at home)

*Abbreviations*: *ARC EM* Applied Research Collaboration East Midlands, *AT* Austria, *BE* Belgium, *CDC* Centers for Disease Control and Prevention, *CoI* Conflict of interest, *COVID* Coronavirus disease, *GER* Germany, *ICU* Intensive care unit, *IT* Italy, *NHS* National Health Services, *NICE* National Institute for Health Care Excellence, *NIHR* National Institute for Health Research, *NL* The Netherlands, *NR* Not reported, *Pts.* Patients, *RCGP* Royal College of General Practitioners, *SIGN* Scottish Intercollegiate Guidelines Network, *UK* United Kingdom, *USA* United States of America

## Results

### Included literature

**Fig. 1:**
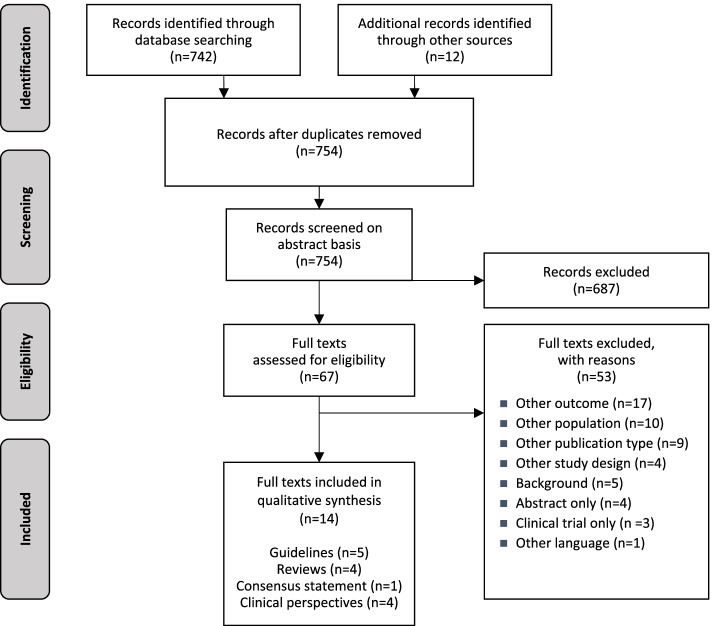
Literature selection process (PRISMA flow diagram)

After deduplication, 754 sources were available for the literature selection on an abstract basis. After the abstract screening, 67 references were considered for further investigation on a full-text basis. A total of 14 references, including five guidelines, four reviews, one consensus statement and four clinical perspectives, met the predefined inclusion criteria and were included to answer the research question. The detailed selection process, including the reasons for excluding 53 full texts, is shown in Fig. 1.

The five included guidelines were from the UK [5, 10], the United States of America (USA) [11], Germany [7] and Austria [12]. In four out of the five guidelines, the recommendations were based on the consensus of expert opinions [5, 7, 10, 12]. The four included reviews were from the UK [2, 13] and USA [14], and one review discussed several European countries, including the UK, Germany, Italy and Belgium [3]. Only one review reported that the recommendations were evidence-based [2]. The five included expert papers were from the UK [15, 16], the Netherlands [17] and Germany [18, 19]. Table 1 presents more detailed characteristics of the 14 included references.

### Recommendations about possible long COVID healthcare pathways

#### First points of contact

##### Primary care

In the included literature, it was recommended that the majority of long COVID-related healthcare should take place in primary care; however, including some differences in the served patient groups between countries. For example, in Austria and Germany, all long COVID patients, including former hospitalized and non-hospitalized COVID-19 patients, are advised to go to the general practitioner (GP) for a first clinical assessment [7, 12]. In contrast, in the United Kingdom (UK), former hospitalized COVID-19 patients, who suffer from ongoing or new symptoms after 12 weeks of discharge, can go to secondary care outpatient departments or primary care facilities for a clinical assessment [5, 10, 15].

Nevertheless, most of all, the GPs or respective healthcare professionals in primary care centres should carry out the primary assessment, including a comprehensive clinical history, the examination of persistent physical, cognitive, psychological and psychiatric symptoms, and functional abilities. Thereby, standard operating procedures may be helpful to identify symptoms that are likely to be caused by the SARS-CoV-2 infection [7, 10–13, 15]. For example, the GPs or respective healthcare professionals in primary care centres can rely on different questionnaires and scales while formulating a diagnosis. Possible questionnaires and scales are listed in Table 2 [5, 7, 11, 16].

**Table 2:** Possible questionnaires and scales for the diagnosis of long COVID

Questionnaire/Scale	Aim	Link
**The EQ-5D**	Assessment of the generic health status of the patients.	https://bit.ly/3v8KRmn
**The Short-Form 36**		https://bit.ly/350qSeT
**The Klok Scale**	Assessment of long COVID-related individual distress and level of impairment.	https://bit.ly/3BGGqjH
**The Newcastle Post-COVID Syndrome Follow-up Screening Questionnaire**	Identification of patients who may benefit from a comprehensive multidisciplinary assessment if symptoms persist for ten to 12 weeks after the acute infection.	https://bit.ly/3HhAHSU
**The COVID-19 Yorkshire Rehabilitation Screening Tool**	Identification of patients who are experiencing problems related to the recent illness with COVID-19	https://bit.ly/3v5w5N6
**The Hospital Anxiety and Depression Scale**	Assessment of anxiety and/or depression.	https://bit.ly/3ptjTlR
**The Patients Health Questionnaire 9**		https://bit.ly/3JKbIZR
**The General Anxiety Disorder 7**		https://bit.ly/3sTDk86
**The Depression Anxiety Stress Scale 21**		https://bit.ly/3t1Nq6L
**The Medical Research Council Dyspnoea Grading Scale**	Measurement of breathlessness.	https://bit.ly/3v357Wq
**The Montreal Cognitive Assessment**	Tool for cognitive screening.	https://bit.ly/3h4ildc

*Abbreviations*: *COVID* Coronavirus disease

In addition, within the primary clinical assessment, it should be differentiated between long COVID symptoms due to organ damage and functional disorders. Furthermore, existing comorbidities, other differential diagnoses, and the socio-economic circumstances of the patients need to be considered [7, 10–13, 15].

##### Secondary care

In some countries (e.g. the UK, USA, Netherlands), the secondary care sector can be recommended as the first point of contact for patients who had been hospitalized during the acute SARS-CoV-2 infection. Hence, follow-up consultations of the patients’ condition can be planned, for example, phone or video consultations by a healthcare professional from secondary care. These consultations should include checking for new or ongoing symptoms and ruling out life-threatening symptoms or other non-COVID-19-related conditions. In addition, it should be assured that the patients had been discharged to the appropriate setting (e.g. home, rehabilitation centre, nursing home). The timing of the first follow-up visit after hospital discharge varies between the countries: In the USA, it should take 6 to 8 weeks [11], while in the Netherlands, it is recommended within 1 to 2 weeks of hospital discharge [17].

In the UK, services also differentiate between former hospitalized COVID-19 patients in general and patients who had been treated in an intensive care unit (ICU) or high dependency unit (HDU). For the latter, the first multidisciplinary assessment of rehabilitation needs should already take place at the point of a step down to other inpatient facilities. Inpatient rehabilitation should begin as soon as the patient is capable of it. After the inpatient rehabilitation and discharge from the hospital, an assessment of the patients’ ongoing needs is recommended, including appropriate community service referrals if needed. Subsequently, a multidisciplinary clinic re-assessment should be undertaken at 4 to 6 weeks post-discharge, including referral if required, e.g. to rehabilitation or mental health services. If the patients continue to improve further, the following assessment is recommended 12 weeks after the hospital discharge [10].

#### Possible further referrals

##### Acute services

If severe, possibly life-threatening symptoms are identified during the first clinical assessment, and the patients should be referred to acute services [5, 7, 10, 11].

##### Specialized long COVID outpatient assessment clinics

In some countries (e.g. UK, GER, AT), patients with no acute or life-threatening complications but with more complex, possibly SARS-CoV-2-related symptoms should be referred to so-called specialized long COVID outpatient assessment centres/clinics. In such outpatient centres/clinics, a second assessment of the patients’ clinical history and current health status is recommended. Thereby, the patients are provided access to multidisciplinary teams, including professionals of, e.g. neurology, psychiatry, psychosomatic, cardiology, pneumology, rheumatology, otorhinolaryngology, dermatology and/or endocrinology. Suppose the assessment results in the need for further assessments and/or therapies. In that case, the centres/clinics can refer the patients to appropriate services, such as specialists of specific disciplines or multidisciplinary rehabilitation programmes. Some of the clinics/centres may also offer treatment options themselves. The timing of a referral to a specialized long COVID outpatient assessment clinic/centre is recommended at any time from 4 weeks after the acute infection, but mostly if symptoms last for more than 12 weeks [5, 7, 10, 11].

##### Specialists

In all investigated countries (UK, USA, NL, GER, AT), patients with a dominant long COVID symptom, e.g. a specific organ dysfunction, should be referred to the relevant specialist, e.g. pulmonologists, cardiologists, neurologists and psychologists. The referral can come directly from the physician who did the first clinical assessment or from specialized long COVID outpatient assessment clinics after the second assessment. The timing of the referral should be based on the individual patients’ needs and the discretion of the assessing clinician; however, mostly it happens if symptoms persist for more than 12 weeks. Subsequently, the specialists can also make further referrals if needed, e.g. to appropriate rehabilitation programmes or other care offers, such as community nursing, to support the patients and wider family members with the treatment process [5, 7, 10–12, 14–16].

##### Rehabilitation

Furthermore, in all investigated countries (UK, USA, NL, GER, AT), there is the option to refer the long COVID patients to multidisciplinary inpatient, partial inpatient or outpatient rehabilitation programmes if necessary. The referrals can be performed directly by the physicians who made the first clinical assessment, specialists, or specialized long COVID outpatient assessment clinics [2, 7, 10–12, 16]. The timing of the referral depends on the severity of the symptom(s). For example, as already mentioned, early rehabilitation should be offered to ICU and HDU patients during the hospital stay if they are capable of it. For patients with mild to moderate symptoms, rehabilitation is usually indicated if the symptoms last for more than 12 weeks [10, 11]. Overall, rehabilitation for long COVID patients should be patient-centred and tailored to the patients’ individual needs, given the wide variety of symptoms and the possible presence of comorbidities. The programmes should also be multimodal and include some of the following elements [2, 3, 5, 7, 10–12, 15–17]:

- Physical elements include, for example, pneumological/cardiological rehabilitation, physiotherapy, speech and language therapy and/or muscle-strengthening programmes, especially for patients who had been treated at the ICU.
- Cognitive elements include physiotherapy and exercise for patients with motor deficits and support in restoring the cognitive function or, if not possible, in developing new ways of organizing information.
- Psychological elements include high-intensity psychological interventions from clinical psychologists, psychiatry, and/or psychological therapies.
- Lifestyle components, such as advice on nutrition, sleep and stress reduction.

In addition, guidelines suggest that the rehabilitation programmes for long COVID patients have a broader scope than usual rehabilitation programmes. For example, a particular focus should be put on the return to work, as a significant proportion of the long COVID patients are in their working age [17]. Furthermore, the programmes should be adapted to the divergent needs of long COVID patients. For example, the concept of “pacing” was named as one of the most important aspects of a long COVID rehabilitation. This concept enables patients to manage their physical, cognitive and emotional energy within their limits through careful planning. Thus, it has been suggested that fixed incremental increases in physical exercise, as with the usual physical rehabilitation, should not be used in long COVID rehabilitation [2, 11–13, 15, 16, 18].

##### Community care networks and non-medical health professionals

Depending on the needs of the patients, they can also be referred to community care networks by their GPs, specialists or specialized long COVID outpatient assessment clinics. These networks involve, for example, community nurses, nursing homes or healthcare hotels [10, 14].

Moreover, in many countries (e.g. UK, GER, AT), patients with a mild to moderate symptom treated by one specific discipline can also be referred to outpatient non-medical health professionals, such as physiotherapists and psychotherapists, occupational therapists, speech therapists or nutritional counselling. Besides, these services can be suggested for patients with more severe symptoms in addition to other treatments (e.g. respiratory physiotherapy) [5, 7, 11, 12].

##### Suggested self-management

**Fig. 2:**
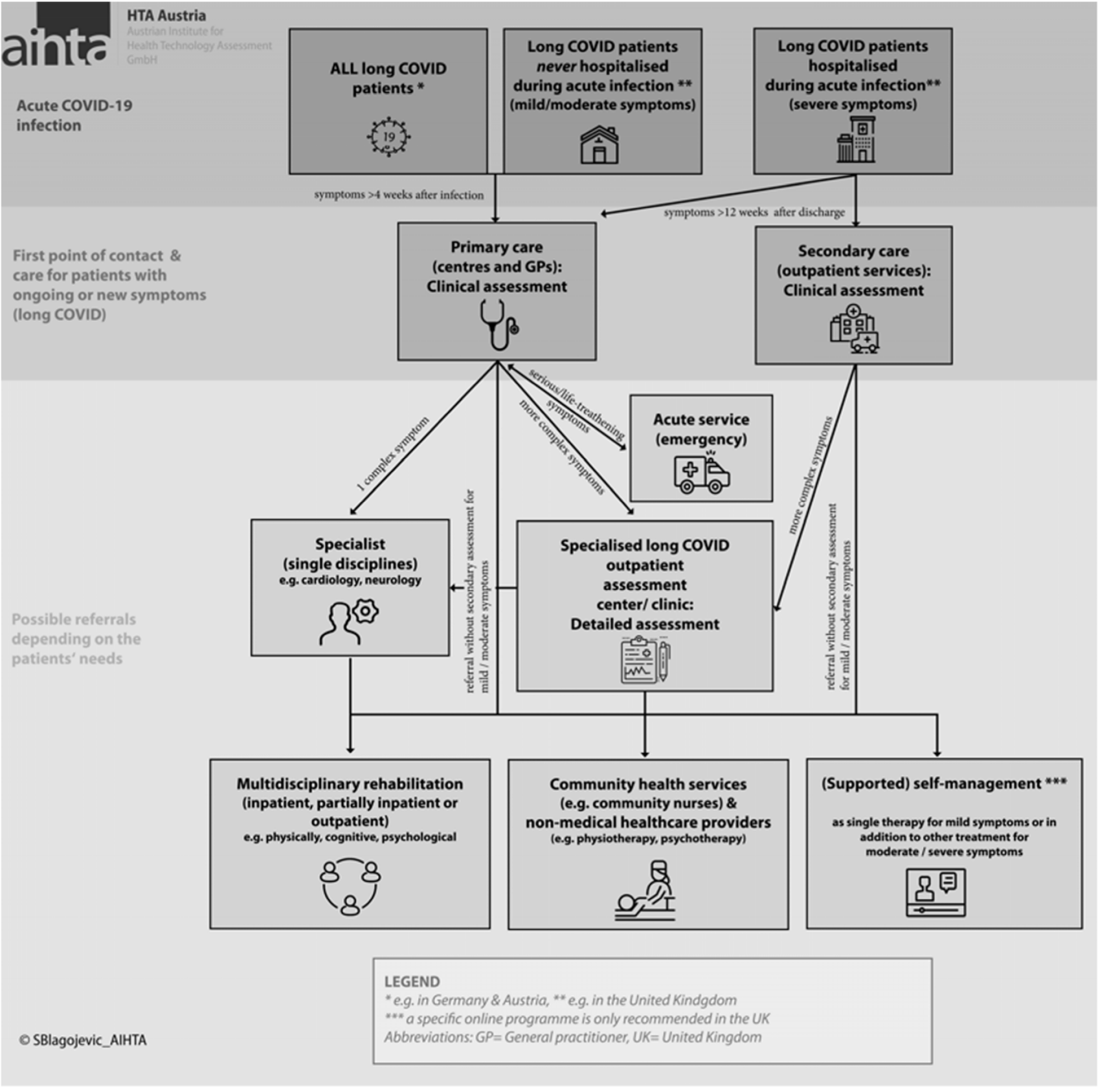
Possible long COVID healthcare pathways (original figure)

Another critical aspect of the investigated recommendations was to advise the patients on how to self-manage their symptoms in addition to other treatments. This can include information about the self-check of clinical parameters (e.g. by oximetry) or self-monitoring by documenting the changes in health conditions and symptom severity (e.g. in a diary). In addition, recommendations about improving general well-being through an appropriate diet, enough sleep or stress reduction were mentioned. Furthermore, supported online programmes can be offered to the patients when available [2, 3, 5, 10, 11, 15, 16]. For example, in the UK, an online self-management programme, the Your COVID-19 Recovery Platform, for long COVID patients was introduced by the National Health Services (NHS). This online programme includes, among other things, a chat where the patients can directly contact healthcare professionals or just join the community forum. To receive access to this online programme, the patients need a referral from a healthcare professional [20]. An overview of the possible long COVID healthcare pathways for adult patients is presented in Fig. 2.

### Further recommendations for practising physicians regarding decision-making and communication

As recommended in the included literature, empathy towards patients, a holistic and patient-centred approach, and shared decision-making between healthcare professionals and patients should be key factors in long COVID healthcare [3, 5, 10–12, 16]. For example, the NHS provides training programmes about “personalized care” for health and care staff by offering access to the Personalized Care Institute. This institute provides high-quality eLearning and training resources in this regard [10].

Furthermore, it is recommended that healthcare professionals constantly update themselves on evolving guidance on long COVID management to inform the patients with the latest information on the disease itself, the possible recovery periods, and therapies [10]. In particular, information about self-management strategies and symptoms when to better look out for professional help should be part of the provided information to the patients [5]. However, in this regard, finding the right balance in the detail of the provided information to avoid unnecessary anxiety and uncertainty in patients, e.g. through overdiagnosis, is crucial [11].

Besides, good communication between healthcare professionals and the patients presents another important aspect in long COVID healthcare. This includes that the practising physicians should consider and minimize possible health inequalities, such as cultural differences, language barriers, mental health conditions, mobility or sensory impairments, learning disabilities by offering special support (e.g. providing translated information about long COVID) [5, 10, 12].

## Discussion

### Interpretation of the main results

#### First point of contacts

In most of the included guidance documents, the primary care sector is recommended for the first clinical assessment [5, 7, 10–12, 14, 16]. However, some difficulties can occur at the first point of contact in clinical practice:

Firstly, long COVID patients can experience hurdles in finding the right GP or primary care centre, as in some countries (e.g. AUT, BE), patients are not assigned to one GP [21].

Moreover, in the absence of simple clinical tests, physicians need to rely on other diagnostic measures, like the history of acute SARS-CoV-2 infection or questionnaires and scales, which are not yet validated for long COVID. Therefore, the diagnosis of long COVID is often complicated, and some symptom severity is hard to detect, which makes an objective diagnosis almost impossible [22]. For these reasons, the practicing physicians should receive further information and training about the disease, its diagnosis and management, including symptomatic treatments, watchful waiting for milder symptoms or possible additional referrals [23]. However, due to the constantly evolving new evidence on long COVID, the content of such training would need to be regularly updated. Apart from that, the development of lists of long COVID specialists in the outpatient and inpatient sectors has been proposed in Germany. Such lists should help GPs refer patients to specialized outpatient clinics or specialists [21].

#### Further referrals

After the first clinical assessments, further assessments and/or treatments by the respective specialist, specialized long COVID outpatient assessment clinic or non-medical healthcare provider can be necessary for some patients with one or more complex symptom(s). However, it is crucial to take into account that many referrals involve multiple doctor visits and thus can place an additional physical, emotional and/or financial burden on the patients [11]. Therefore, non-medical aspects, such as the geographical distance between the patient’s residence and the treatment location and private co-payments, especially for non-medical healthcare therapies, need to be considered in the decision about a referral. Alternatively, where appropriate and possible, virtual consultations may be suggested to eliminate additional travel time and costs [7, 11, 12, 16, 18]. Besides, several referrals can also increase the risk of contradictory medical advice, causing uncertainty in the patients [11]. Specific follow-up strategies, such as a practice plan for continuing exercises at home, referral to outpatient non-medical healthcare therapies or support through social workers, are also recommended to minimize possible uncertainties in returning to usual daily activities [24].

#### Self-management

In the analyzed recommendations, advice on self-managing the symptoms was another crucial aspect for long COVID healthcare. However, self-management can also place a lot of responsibility on the patients, which might cause an additional burden to them [25, 26]. In addition, the exchange with other long COVID patients (e.g. within a forum) might also increase anxiety in some patients [22].

#### Decision-making and communication

The included evidence showed that knowledge about the possible point of services and long COVID specific care elements is essential. In addition, good communication between healthcare professionals and patients is also critical in long COVID care.

#### Economic considerations

From an economic point of view, the organization of long COVID healthcare structures triggers concerns about resource capacities. For example, long waiting lists might exist within the healthcare systems for a first appointment in a specialized long COVID outpatient assessment clinic or a place in a rehabilitation programme. One reason for such capacity shortages might be that specialized rehabilitation centres are sparse in some countries. Access is classically restricted to patients with other indications than long COVID, such as stroke or head trauma. Another reason is patients’ preferences: the geographical distance to the care facility might hinder a patient and eventually cause refusal of a rehabilitation place. Consequently, more and more private providers appear on the market offering complete all-around packages with shorter waiting times and various destinations. Thereby, the two-tier healthcare system is further enhanced [24].

Moreover, there is a concern if enough GPs are available, considering the expected increase in long COVID patients and the GPs’ primary role in long COVID healthcare. Consequently, it should also be discussed in the future if patients should have the possibility to contact directly, for example, health professionals, such as physiotherapists or psychotherapists, in a first step to ease the burden on the GPs.

In addition, the overall costs and the sustainability of long COVID healthcare remain unclear and need to be assessed in the future.

### Limitations of the present scoping review

This scoping review addresses a topic of high public interest. However, some limitations persist due to the applied methods, the restricted inclusion criteria, and the included evidence quality.

The literature searches were limited to one systematic search at the end of April 2021 and biweekly unsystematic hand searches until August 2021. Due to the rapidly emerging evidence on this topic, not all relevant publications could be identified. For example, a Canadian systematic review about long COVID care models from June 2021 was not included in the present scoping review because its inclusion criteria differed from the present review’s [27]. Besides two references about long COVID care models [10, 13], which were also included in this review, it contained ten additional references. In line with the results of this review, the Canadian study identified primary care, specialized clinics and rehabilitation services as central aspects of long COVID healthcare. In addition, the Canadian review suggested a centralized referral system [27].

Furthermore, in the narrative synthesis of the extracted data, terminologies of the original references were standardized. Thus, minor differences in the meaning of some terminologies may not be visible at a glance.

Besides, the present scoping review only focused on adult long COVID patients in general. However, as children and adolescents or vulnerable groups, such as socially disadvantaged persons, may require different healthcare professionals and healthcare structures, this needs to be investigated separately in the future.

Furthermore, it only addressed *healthcare pathways* for long COVID patients. Detailed long COVID treatment recommendations were not part of this review. However, other reviews started focusing on recommendations for specific long COVID treatments [15, 28]. Another review also reported the first effectiveness studies of long COVID therapies [29].

Moreover, only German or English information could have been considered to answer the research question. Therefore, relevant information on healthcare pathways may have been overlooked.

Apart from that, it needs to be pointed out that the included literature is limited in its nature due to the novelty of this topic and the associated urgency for research. Most of the recommendations in the included guidelines were preliminary and based on expert consensus. Moreover, the included reviews were limited in their methodology and did not present systematic reviews. Furthermore, given the sparsely available evidence on long COVID healthcare pathways at the time of conducting this scoping review, expert papers in the form of consensus papers or clinical perspectives were also taken into account, even if they show the lowest level of evidence and are associated with a high risk of bias. The expected limited quality of evidence might have an unintended impact, as Stamm et al. summarised: “An insufficient consideration of appropriate methodologies in the guideline development process could lead to misleading information, uncertainty among the professionals, and potentially harmful actions for patients” [30].

## Conclusions and preview

This scoping review showed recommendations for long COVID healthcare pathways from preliminary guidelines, reviews and expert papers. This weak evidence-base of the existing guidelines calls for an update with more rigorous data and guideline development processes.

Furthermore, the exact epidemiology of long COVID is still not clarified. However, these epidemiological data are urgently needed for efficient long COVID healthcare planning and hence, to reduce the likelihood of overwhelming the medical system or establishing inappropriate or excess care facilities [3, 31]. For this reason, the collection of real-world data on the long COVID surveillance is recommended [32]. For example, long COVID registries could be set up to collect data on the duration and the severity of persistent symptoms after acute SARS-CoV-2 infection in different sub-groups [3, 31]. Thereby, patients should be ideally involved in the research planning to ensure a patient-centred approach [32–34]. Last but not least, the collection of such real-world data would benefit from international cooperation, as these data are disease-specific and might not significantly differ between countries [3].

Moreover, there is little evidence about the effectiveness and safety of possible treatment options for long COVID patients. Therefore, more high-quality clinical trials and systematic reviews summarising the results of clinical trials are needed to investigate the safety and efficacy of potential treatments and interventions for long COVID patients (e.g. the impact of the COVID-19 vaccine on long COVID, preferred type of rehabilitation or specific treatments for children and adolescent with long COVID) [2, 3, 7, 31].

Finally, this scoping review only focused on possible healthcare pathways for adult long COVID patients. In the future, further reviews need to address possible healthcare pathways for children and adolescents, as their needs might differ from adult patients.

## Data Availability

The complete data analyzed during the current study are available from the corresponding author on reasonable request.

## Abbreviations

ARC EM: Applied Research Collaboration East Midlands
AT: Austria
BE: Belgium
CDC: Centers for Disease Control and Prevention
CoI: Conflict of interest
COVID: Coronavirus disease
e.g.: For example
GER: Germany
GP: General practitioner
HDU: High dependency unit
INAHTA: International Network of Agencies for Health Technology Assessment
ICD-10: International Classification of Diseases
ICU: Intensive care unit
IT: Italy
NHS: National Health Services
NICE: National Institute for Health and Care Excellence
NIHR: National Institute for Health Research
NL: The Netherlands
NR: Not reported
PRISMA: Preferred Reporting Items for Systematic Reviews and Meta-Analyses
Pts.: Patients
RCGP: Royal College of General Practitioners
SARS-CoV-2: Severe acute respiratory syndrome coronavirus 2
SIGN: Scottish Intercollegiate Guidelines Network
UK: United Kingdom
USA: United States of America
WHO: World Health Organisation
